# Single cell fluorescence imaging of glycan uptake by intestinal bacteria

**DOI:** 10.1038/s41396-019-0406-z

**Published:** 2019-04-01

**Authors:** Jan-Hendrik Hehemann, Greta Reintjes, Leeann Klassen, Adam D. Smith, Didier Ndeh, Carol Arnosti, Rudolf Amann, D. Wade Abbott

**Affiliations:** 1 Max Planck-Institute for Marine Microbiology, 28359 Bremen, Germany; 2 Center for Marine Environmental Sciences, University of Bremen (MARUM), 28359 Bremen, Germany; 3 Lethbridge Research and Development Centre, Agriculture and Agri-Food Canada, 5403-1st Avenue South, Lethbridge, AB T1J 4B1 Canada; 4 Institute for Cell and Molecular Biosciences, The Medical School Newcastle University, Framlington Place, Newcastle upon Tyne, NE2 4HH UK; 5 Department of Marine Sciences, University of North Carolina, Chapel Hill, NC USA

**Keywords:** Metabolomics, Biological techniques

## Abstract

Microbes in the intestines of mammals degrade dietary glycans for energy and growth. The pathways required for polysaccharide utilization are functionally diverse; moreover, they are unequally dispersed between bacterial genomes. Hence, assigning metabolic phenotypes to genotypes remains a challenge in microbiome research. Here we demonstrate that glycan uptake in gut bacteria can be visualized with fluorescent glycan conjugates (FGCs) using epifluorescence microscopy. Yeast α-mannan and rhamnogalacturonan-II, two structurally distinct glycans from the cell walls of yeast and plants, respectively, were fluorescently labeled and fed to *Bacteroides thetaiotaomicron* VPI-5482. Wild-type cells rapidly consumed the FGCs and became fluorescent; whereas, strains that had deleted pathways for glycan degradation and transport were non-fluorescent. Uptake of FGCs, therefore, is direct evidence of genetic function and provides a direct method to assess specific glycan metabolism in intestinal bacteria at the single cell level.

Polysaccharides are an important food component for intestinal microbes and support genetic diversity within the gut microbiome [1]. The chemical energy stored in structurally diverse glycans from plants and fungal cell walls provide an extensive resource landscape for microbial adaptation [2]. When presented to the intestinal microbiome of animals, these glycans selectively enrich bacteria endowed with cognate glycan degrading pathways [2, 3]. The fermented end-products of glycan utilization by gut bacteria are short chain fatty acids, which are host absorbable secondary metabolites and represent approximately 10% of human caloric intake [4]. Therefore, conversion of indigestible glycans into compounds for host utilization is an essential symbiotic role for gut microbes that have evolved to occupy dynamic nutrient niches. Adaptation to specific dietary glycans unfolds on time scales relevant to a human life span [5]. This suggests that glycans, and functional foods in general, can be harnessed as personalized interventions to modulate the function of the gut microbiome in ways that benefit human health, such as selecting for the growth of desired taxa [6].

Underlying resource partitioning within the gut microbiome are genome encoded pathways specific for the degradation of chemically distinct glycans. These pathways are encoded in operons known as polysaccharide utilization loci (i.e. PULs), and contain genes required for sensing, depolymerizing, and transporting glycans [3, 7]. Because PULs contribute to fundamental digestive processes within the host intestine, improved research methods to establish metabolic abilities present within the microbiome and to explore how microbes respond to dietary interventions are urgently needed. More specifically, although biochemical characterization of PULs from cultivable isolates has made remarkable progress in defining the molecular basis of PUL function and the broad scale diversity of PULs can be assessed with metagenomics, the field is lacking methods that rapidly assign metabolic phenotypes to genotypes on the single cell level within a microbial community.

Marine bacteria have recently been shown to selectively import fluorescently labeled polysaccharides into their periplasm [8]. In these experiments substrate-based staining was combined with single cell identification by fluorescence in situ hybridization. Here we chose the gut bacterium *Bacteroides thetaiotaomicron* VPI-5482 (hereafter *B. theta*) as a model organism to ascertain whether glycans found within human diets can be fluorescently labeled and used to visualize selective glycan metabolism. *B. theta* consumes yeast α-mannan (YM) and rhamnogalacturonan-II (RGII) (Fig. 1a, b) with proteins that are encoded by specifically adapted PULs (SI Fig. 1) [9, 10]. Remarkably, despite RGII’s extensive structural complexity (SI Fig. 1D) *B. theta* was shown to cleave all but one of the 21 distinct linkages and utilize all but four of the liberated monosaccharides for energy [10]. These complex glycans are utilized by *B. theta* through a “selfish mechanism” [9], a feeding strategy that is thought to limit the distribution of “public goods” [3, 11] to other members of the community. A hallmark of the selfish uptake mechanism is that complex products generated by extracellular CAZymes are selectively imported and the majority of saccharification occurs within the confines of the periplasm. This feeding strategy underpins that Bacteroidetes SusC/D-like TonB-dependent transporters can accommodate large, energy-rich polysaccharides for cellular metabolism.

**Fig. 1:**
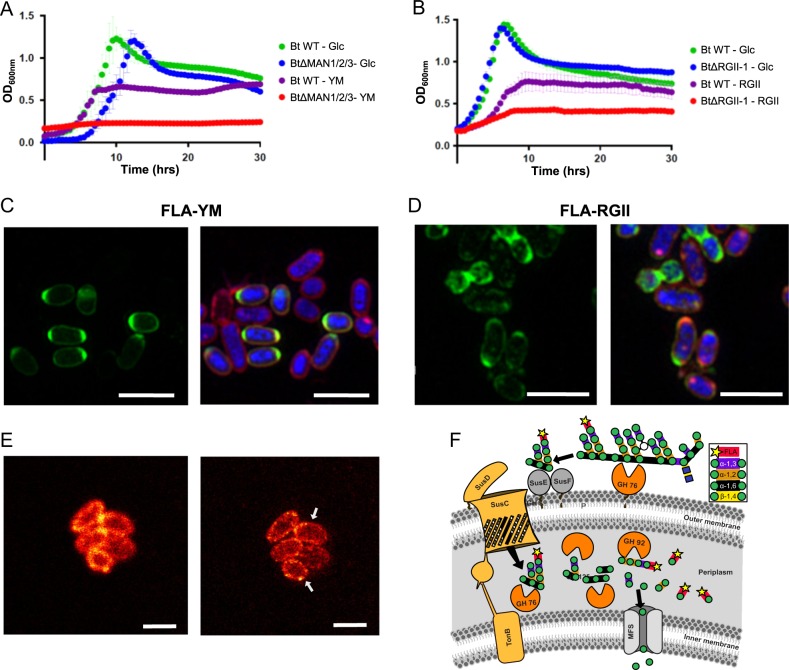
Uptake of fluorescent glycans by *B. theta*. **a** Anaerobic growth profiles of *B. theta* and BtΔMAN1/2/3 cultured on YM and glucose. **b** *B. theta* and BtΔRGII cultured on RGII and glucose. Growth curves display the mean of five independent replicates reported every 30 min; error bars represent s.d. **c** Uptake of FLA-YM and **d** FLA-RGII by *B. theta*. Super resolution structured illumination (SR-SIM) images of representative cells at 72 h. Left panels are fluorescent signals from FLA-probes; right panels are overlays of DAPI, FLA-probes, and Nile Red. Scale bars represent 2 μm. **e** Uptake of FLA-YM by *B. theta* visualized by confocal laser scanning microscopy image (CLSM, left) and stimulated emission depletion microscopy image (STED, right). **f** The illustration represents the selfish mode of yeast mannan metabolism by *B. theta*, adapted from [9]. Following limited cleavage by surface exposed GH76 enzymes, product uptake is mediated by SusC/D-like proteins. The majority of saccharification occurs in the periplasm and is mediated by carbohydrate active enzymes spedific for the hydrolysis of α-mannosyl linkages (e.g. GH92, GH76, and GH125). During this process FLA is transported into the periplasm, where it accumulates and can be visualized by super-resolution microscopy. Scale bar represents 1 µm

To determine if selfish metabolism in *B. theta* could be leveraged for imaging, YM and RGII were conjugated at free diols with 6-aminofluorescein (FLA) using a cyanogen-bromide activation chemistry [12, 13]. The products of these reaction are fluorescent glycan conjugates (FGCs) FLA-YM and FLA-RGII. *B. theta* was cultured on unlabeled YM and RGII to metabolically activate the cells, incubated with FLA-YM or FLA-RGII, and then visualized by epifluorescence and super-resolution microscopy. Cells treated with FGCs displayed intracellular accumulation of fluorescent signal (Fig. 1c, d); whereas, cells incubated with unlabeled glycan did not display any fluorescence (SI Fig. 2). We observed that FGC uptake was time-dependent, with some *B. theta* cells showing rapid incorporation of fluorescent signal (after minutes) and peaking at 24 and 72 h for FLA-YM and FLA-RGII, respectively (SI Figs. 2 and 3). These observations highlight that uptake rates of FGCs can differentiate between PULs, which may reflect the structural complexity of the imported glycan; and FGCs represent potent tools to detect and study differential mechanisms of glycan uptake and metabolism by Bacteroidetes. The differences in uptake between FLA-YM and FLA-RGII were somewhat surprising as the cells were not actively dividing at this stage of the growth, and previously, xylan and laminarin displayed similar uptake kinetics in marine Bacteroidetes [8]. Therefore, this effect may result from the unique, highly branched structures of YM and RGII (Fig. 1b, d) (SI Fig. 1B, D). Alternatively, stochastic additions of FLA conjugations may result in distinct labeling density and positional chemistries, which could effect transport efficiency [12].

Although experiments were carried out with pure cultures, we observed heterogeneity in the extent of cell fluorescence for both FLA-YM and FLA-RGII (Fig. 1c, d). Some cells were highly fluorescent while others did not show any signal. This heterogeneity might indicate that uptake efficiency differs between cells within a population, which has been frequently observed for microbes [14]. In this light, FGCs appear uniquely suited to query heterogeneity of carbohydrate metabolism between individual cells. Overlays of the fluorescent signals for cellular DNA and the FGCs revealed that FGCs concentrated around the proximity of the cell forming a halo. Additional staining with Nile Red, a dye specific for the membrane lipid bilayer, demonstrated that green and red fluorescence co-localized suggesting that the glycan accumulates within the periplasm (Fig. 2a, b). Previously, accumulation of FGCs in the periplasm was reported in marine Bacteroidetes [8] and is also supported by stimulated emission depletion (STED) microscopy (Fig. 1e) and enzyme protection assays (SI Fig. 4). These patterns of FLA-YM visualization are consistent with the selfish mode of glycan metabolism previously described for YM consumption by *B. theta* (Fig. 1f, [9]).

**Fig. 2:**
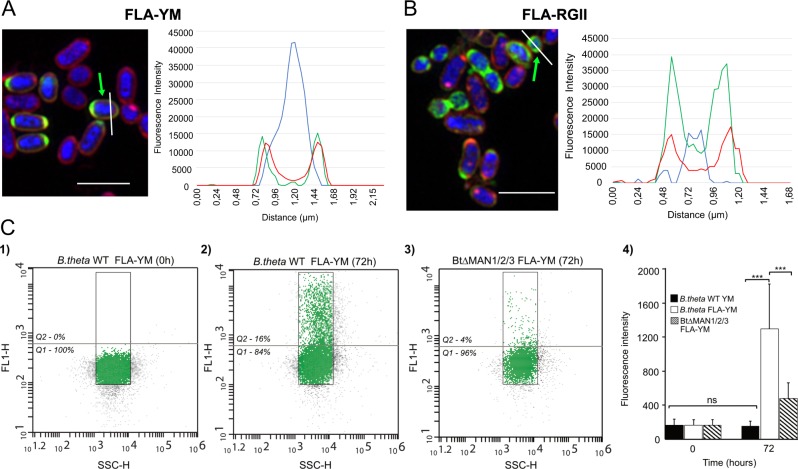
Differential uptake of FGCs by individual cells in a population. **a** Cellular localization of DNA (DAPI), membrane (Nile Red), and FLA-YM or **b** FLA-RGII after 72 h. Left panel: SR-SIM single cell images of *B. theta* cells labeled with DAPI, Nile Red, and FGC. Cells that were selected for fluorescence intensity line profiling are indicated with a green arrow and white line for profile. Right panel: Line profiles indicating fluorescence intensity at different wave lengths. Blue: DAPI; Green: FLA; and Red: Nile Red. **c** Quantification of FLA-YM uptake by *B. theta* and mutant *B. theta* (BtMAN1/2/3) strains over time using flow cytometry. (**1**) *B. theta* grown on YM shows no background fluorescence (FL1-H) signal. (**2**) *B. theta* shows significant increase in cellular fluorescence after 72 h incubation with FLA-YM. (**3**) In a *B. theta* strain with all three YM PULs deleted (BtΔMAN1/2/3) showed a low increase in fluorescence after 72 h incubation with FLA-YM. Green gating shows main cell population (*n*(gate) = 10,000 events). Gray line indicates percentage of cells above (Q2) and below (Q1) minimum FL1-H threshold, gated according to T0 negative sample. (**4**) Bar graph showing the mean fluorescence intensity of *B. theta* (white) and BtΔMAN1/2/3 (dashed) incubated with YM-FLA and a control (*B. theta* incubated with unlabeled YM, black) over time. *N* = 8500 and error bars = standard deviation. Statistical differences were calculated by Welch’s *t*-test (ns: no significant difference, ***P *<* 0.001)

Next, we investigated to what extent FGC uptake relies on the presence of specific PULs. Mutant *B. theta* strains with targeted PUL deletions for YM (BtΔMAN1/2/3) [9] and the major RGII PUL (BtΔRGII) [10] were incubated with FLA-YM and FLA-RGII, respectively (SI Fig. 2). In both cases, PUL removal ablated FGC uptake and fluorescence. This could also be verified, for YM, by FLA signal quantification, using flow cytometry (Fig. 2c), which showed that there was a significant difference in signal intensity between the mutant and WT strains. These results confirmed that glycans are modified and/or transported by proteins encoded within the PULs [7, 15]. In the BtΔRGII strain, there was low-level residual staining for RGII substrates (SI Fig. 2B), which suggests the intact orphan SusC/D-like wine-RGII-specific transporter, BT1682/BT1683, can still import FLA-RGII at basal levels and that it accommodates minimally processed forms of RGII (SI Fig. 1B [10]). Therefore, FGCs can also be used to study preferential PUL hierarchy for engineered systems with multiple loci.

## Conclusion

Glycan utilization by intestinal Bacteroidetes is critical for digestion of dietary carbohydrates. This host–symbiont interaction is facilitated by cognate PUL systems that endow bacterial strains for consumption of specific glycans. Despite the remarkable progress that has been made in cataloging microbiome composition from diverse sources [16] and defining the molecular basis of PUL function [2], the field still is lacking methods to rapidly assign metabolic phenotypes to genotypes in microbial communities at the single cell level. FGCs provide tools for rapid and selective study of glycan–microbe interactions, and will assist in deciphering the mechanisms driving microbiome responses to dietary interventions [17].

## Materials and methods

### Generation of FGCs

Mannan from *Saccharomyces cerevisiae* (YM) prepared by alkaline extraction was purchased from Sigma (St. Louis, MO, USA; M7504). Wine RGII, purified as described previously [18]. Activation and labeling of YM and RGII was carried out as described in ref. [13], with slight modifications. Briefly, 40 mg of polysaccharide was solubilized in 2 mL distilled H_2_O (dH_2_O). To activate the polysaccharide, 30 mg of cyanogen bromide (CNBr; 97%; Sigma C91492) in 350 µL dH_2_O was added, with subsequent 20 µL additions of 0.25 M NaOH to maintain a pH > 9.5 for 6 min. Immediately after activation, the reaction solution was loaded onto a gel permeation chromatography column to separate excess CNBr from activated polysaccharide. The column (30 × 1 cm), filled with Sephadex G-50 gel, was connected to a multistatic peristaltic pump (Bio-Rad BioLogic LP), which set a flow rate of 1.0 mL min^−1^ with 0.2 M sodium tetraborate decahydrate (≥99.5%, pH 8.0; Sigma S9640) as the mobile phase. Eluate was monitored by UV absorbance (280/254 nm) and recorded using Bio-Rad LP Data View software. The void volume was collected directly into vials containing 2.0 mg fluoresceinamine isomer II (FLA; ~95%; Sigma O7985). Each vial was covered in aluminum foil to prevent decay and incubated for 24 h at room temperature. The labeled products were then concentrated and separated from excess FLA using Sartorius Vivaspin 15R spin columns (5000 molecular weight cut off; product no. VS15RH11). Columns were centrifuged (rcf: 210 × *g*) at ambient temperature. Filtrate was removed and the sample was washed with 1 mL, dH_2_O. This process was repeated until the filtrate was clear of any visible orange color that is a result of FLA in solution. The purified samples were flash frozen, lyophilized, and stored at ~4 °C in the dark.

### FGC uptake by *Bacteroides thetaiotaomicron*

Five milliliters of supplemented brain heart infusion (BHI; Difco) medium was inoculated with *B. theta*, BtΔMAN1/2/3 [9], or BtΔRGII [10]. Cultures were grown under anaerobic conditions (85% N_2_, 10% CO_2_, and 5% H_2_) at 37 °C as described previously [19]. Cells were harvested at mid-exponential to late-exponential phase (OD_600nm_ 0.6–1.0), centrifuged (4700 × *g* for 5 min), and washed in 2× minimal media, which contained: NH_4_SO_4_ (8.5 mM), Na_2_CO_3_ (9.4 mM), l-cysteine-free base (4.1 mM), KH_2_PO_4_ pH 7.2 (100 mM), FESO_4_·7H_2_O (1.4 μM), vitamin K_3_ (1 µg mL^−1^), vitamin B_12_ (5 ng mL^−1^), NaCl (15.4 mM), CaCl_2_ (0.24 mM), MgCl_2_·6H_2_O (98 μM), MnCl_2_·4H_2_O (50 μM), CoCl_2_·6H_2_O (42 μM), resazurin (1 µg mL^−1^), and histidine/hematin solution (2 µL mL^−1^ v/v; 1.9 μM hematin/200 μM l-histidine; 1000× stock solution). The washing step was repeated two more times, with the final resuspension in 2 mL 1× MM with 0.5% YM (WT), 0.5% RGII (WT), 0.5% glucose + YM (*B. theta* and BtΔMAN1/2/3), or 0.5% glucose + RGII (*B. theta* and BtΔRGII) as the sole carbon source (unlabeled). Cultures were incubated overnight.

After 20–24 h of incubation, the cells were centrifuged and washed three times as explained above. The final pellet was resuspended in 2 mL 2× MM and was used as the inoculum. 150 µL of each inoculum was added to 1 mL 0.2% polysaccharide (made by mixing 500 µL 0.4% polysaccharide with 500 µL 2× MM). After the washes, the overnight cultures were inoculated into the appropriate polysaccharide substrate, as follows: *B. theta* incubated in 0.5% RGII was inoculated into both 0.2% RGII and 0.2% FLA-RGII. *B. theta* incubated in 0.5% YM was inoculated into both 0.2% YM and 0.2% FLA-YM. The BtΔRGII mutant incubated in 0.5% glucose + RGII overnight was inoculated into both 0.2% RGII and 0.2% FLA-RGII. The BtΔMAN1/2/3 mutant incubated in 0.5% glucose + YM was inoculated into both 0.2% YM and 0.2% FLA-YM. Culture tubes were wrapped in aluminum foil to prevent decay of fluorescence.

### Cell fixation

Twenty microliters of each growth cultures were aliquoted into sterile  2 mL screw cap tubes from the cells and resuspended in 2 mL 2× MM (0 h). FLA-labeled and unlabeled substrates were then added to the activated cells. 40 µL samples of each treatment were taken at three time points: 1 min, 24, and 72 h. The samples were centrifuged (rcf: 1500 × *g*) for 10 min, and the supernatants removed. To fix the cells, pellets were resuspended in 1 mL formaldehyde (Sigma; product no. F8775), diluted to 1% with 1× phosphate-buffered saline (PBS; pH 7.4): 137 mM NaCl, 2.7 mM KCl, 10 mM Na_2_HPO_4_, 1.8 mM KH_2_PO_4_, and incubated overnight at 4 °C. The next day, cells were centrifuged (rcf: 1500 × *g* for 10 min), pellets washed in 1× PBS, and centrifuged a final time. The supernatant was removed and the pelleted cells were stored at 4 °C until further analysis.

### Epifluorescence microscopy

For epifluorescence microscopy fixed *B. theta* cells were filtered onto 25 mm polycarbonate filters (0.2 µm pore size) using a gentle vacuum (<200 mbar). The cells were then counterstained with DAPI and mounted using a Citiflour (Electron Microscopy Sciences, USA) and Vector Shield (Vector Laboratories, Germany) mounting solution (4:1). The cells were visualized on a Zeiss Axioskop 2 motplus fluorescent microscope using the Axiovision software (Zeiss, Germany).

### Super-resolution microscopy

For visualization, 30 μL of fixed cell culture was heat fixed at 40 °C to poly-d-lysine-coated coverslips (#1.5, thickness 0.17 mm). Subsequently, additional salts from the medium are removed by washing the coverslip in MQ.

For super-resolution structured illumination microscopy (SR-SIM) the cells were then counter-stained with 4′,6-diamidino-2-phenylindole (DAPI) (1 ng μL^−1^ WS) and Nile red (2 ng μL^−1^ WS) for 10 and 25 min, respectively. After each stain, the cells are washed in MQ and, subsequently, mounted using a 4:1 Citifluor/VectaShield mounting solution. Cells were visualized on a Zeiss ELYRA PS.1 (Carl Zeiss) using 561, 488, and 405 nm lasers and BP 573-613, BP 502-538, and BP 420–480 + LP 750 optical filters. Z-stack images were taken with a Plan-Apochromat ×63/1.4 oil objective and processed with the software ZEN2011 (Carl Zeiss, Germany). Fluorescence intensity line profiles of individual cells were carried out using the ZEN black software.

For STED and confocal laser scanning microscopy (CLSM) cells were mounted using Prolong Diamond (Thermo Fischer, Germany). The cells were visualized on an Abberior Instrument (Aberrior Instruments GmbH, Germany) using, for FLA-YM detection, a 485 nm excitation laser, 525/50 nm detector and 595 nm STED laser were used, with a pinhole of 70 µm, a pixel size of 20 nm, dwell time 20 µs and nine line accumulation.

### Enzyme protection assays

To determine if FGCs were imported into the cells, an aliquot of FLA-YM was digested by α-mannan-specific enzymes from *B. theta* [9, 20], including three α-mannosidases (BT3781 = α1,6-GH125; BT3782 = BT3990 = α1,2-GH92; BT3991 = α1,3-GH92) and one α-1,6-mannanase (BT3782 = GH76). Digestions were carried out with 0.2% FLA-YM and 1 μM of each enzyme for 1 h at 37 °C in 1X MM (pH 7.2). Digested or undigested FLA-YM was incubated with *B. theta* anaerobically at 37 °C for 24 h. The sample with intact FLA-YM was treated with identical enzyme conditions described above. All samples were fixed in 1% formaldehyde for 1 h at ambient temperature, and washed in 1× PBS buffer. Samples were stored at 4 °C. Cells were analyzed by epifluorescence with constant exposure times (see above).

### Flow cytometry and fluorescence quantification

Cell fluorescence due to FLA-substrate uptake was quantified in all growth cultures using an Accuri C6 flow cytometer (BD Accuri Cytometers) as described previously [8]. The 8-peak and 6-peak validation bead suspensions (Spherotech, Lake Forest, IL, USA) were used as internal references. The FCM output was analyzed using FlowJo v10.4.2 (Tree Star, USA). The FCM files were imported into FlowJo, and the main population (representing single cells) was gated (green) in the FL1-H and SSC-H view. The sample statistics (counts, mean and standard deviation) and raw FL1-H results for each event in the gate (*n* = 10,000) were exported and analyzed using the R software. Statistical differences were calculated by Welch’s *t*-test in R. Additionally the percentage of total events which were above (Q2) or below (Q1) the minimum FL1-H values, gated according to controls events FL1-H was calculated.

### Growth profiling of *B. theta* strains

Five milliliters of BHI media was inoculated from frozen stocks of *B. theta*, BtΔMAN1/2/3, and BtΔRGII. During exponential phase, cells were centrifuged (rcf: 4700 × *g* for 5 min) and washed with 2× MM. The cells were diluted to OD_600nm_~0.15. Falcon 300 µL flat-bottomed 96-well microtiter plates were used for growth curves. Each well was filled with 100 µL filter sterilized substrate (1% glucose, 1% mannose, 1% YM, 1% RG-II), as well as 100 µL of inoculum (*n* = 6), to get a final concentration of 0.5% substrate and 1× MM. 100 µL of sterile water was added to wells with 100 µL culture as negative controls. Positive controls consisted of 180 µL of BHI and 20 µL culture. Breathe-Easy gas-permeable polyurethane membranes (Sigma-Aldrich Z380059) were used to seal the multiwell plates. An Eon microplate reader (Biotek) with Gen5 software (BioTek) was used to measure and record absorbance (600 nm) every 30 min for 48 h. Data was analyzed using GraphPad Prism software.

## Supplementary information

Sup4

Supplementary Figure captions

Sup1

Sup2

Sup3
