# Supplementary material for: Single cell fluorescence imaging of glycan uptake by intestinal bacteria
Source: ISME J. 2019 Apr 1;13(7):1883–9. doi: 10.1038/s41396-019-0406-z (PMC6776043; doi:10.1038/s41396-019-0406-z)
Supplement: Supplementary file 1 — Sup4 [file 41396_2019_406_MOESM1_ESM.pdf]

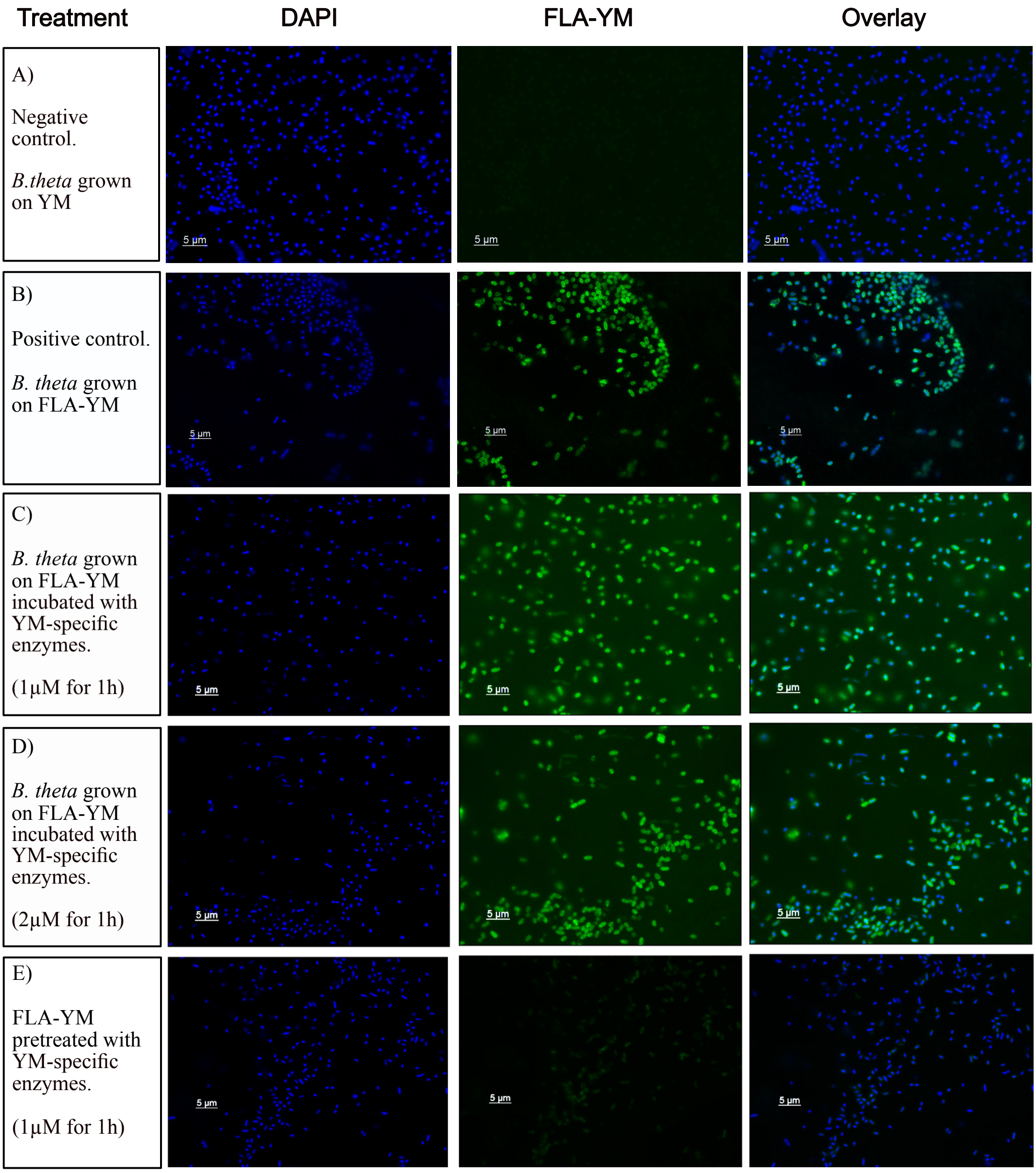

Supplementary Figure 4: Epifluorescence microscopy images of enzyme protection assays. A & B) Negative and positive control showing cells grown on YM and cells incubated in FLA-YM. **C & D)** FLA-YM stained cells incubated in YM specific enzymes. **E)** FGC treated with YM-specific enzymes before incubation with *B. theta*. Exposure times are consistent between images and signal loss represents depletion of FLA-YM. Scale bar = 5 µm.
