# Supplementary material for: Single cell fluorescence imaging of glycan uptake by intestinal bacteria
Source: ISME J. 2019 Apr 1;13(7):1883–9. doi: 10.1038/s41396-019-0406-z (PMC6776043; doi:10.1038/s41396-019-0406-z)
Supplement: Supplementary file 2 — Supplementary Figure captions [file 41396_2019_406_MOESM2_ESM.docx]

**Supplementary Information**

**Supplementary Figure 1:** (**a**) Structure of PUL‐MAN1, 2 and 3, pathways responsible for the utilization of YM by *B. theta*. (**b**) Composition and linkage structure of YM. (**c**) Structure of PUL‐RGII1, 2, and 3 responsible for the utilization of RGII by *B. theta*. Annotated genes and enzyme family are labeled. (**d**) Composition and linkage structure of RGII. Annotated genes and enzyme families are labeled. The gray boxes denote the genes that were removed by mutagenesis. Monosaccharides are depicted using the Consortium for Functional Glycomics nomenclature (18).

**Supplementary Figure 2:** Full panel display of fluorescently labeled *B. theta* mutant strains visualized by super-resolution structured illumination microscopy (SR-SIM). Cells are stained with DAPI (blue), FLA-YM or FLA- RGII (green), and Nile Red (red); and displayed at 0* (true zero), 0 (directly after glycan addition), 24 and 72 hours. (A & B) Wild-type *B. theta* and mutant Bt∆MAN1/2/3 cells labeled with FLA-YM. (C & D) Wild-type *B. theta* and mutant Bt∆RGII cells labeled with FLA-RGII. Size bars = 2 μM.

**Supplementary Figure 3:** Time-dependent uptake of fluorescent glycan conjugates by

*B. theta*. **A**) Change in mean fluorescence intensity of *B. theta* (white) and Bt∆MAN1/2/3 (dashed) incubated with FLA-YM and a control (*B. theta* incubated with unlabeled YM, black) over time (0* (true zero), 1 min (directly after glycan addition), 24 and 72 hours). **B**) Change in mean fluorescence intensity of *B. theta* (white) and Bt∆RGII (dashed) incubated with FLA-RGII and a control (*B. theta* incubated with unlabeled RGII, black) over time (0* (true zero), 1min (directly after glycan addition), 24 and 72 hours). N=8,500 and error bars = standard deviation.

**Supplementary Figure 4:** **Epifluorescence microscopy images of enzyme protection assays.** **A & B)** Negative and positive control showing cells grown on YM and cells incubated in FLA-YM. **C & D)** FLA-YM stained cells incubated in YM specific enzymes. **E)** FGC treated with YM-specific enzymes before incubation with *B. theta.* Exposure times are consistent between images and signal loss represents depletion of FLA-YM. Scale bar = 5 µm.
