# Supplementary material for: Single cell fluorescence imaging of glycan uptake by intestinal bacteria
Source: ISME J. 2019 Apr 1;13(7):1883–9. doi: 10.1038/s41396-019-0406-z (PMC6776043; doi:10.1038/s41396-019-0406-z)
Supplement: Supplementary file 3 — Sup1 [file 41396_2019_406_MOESM3_ESM.pdf]

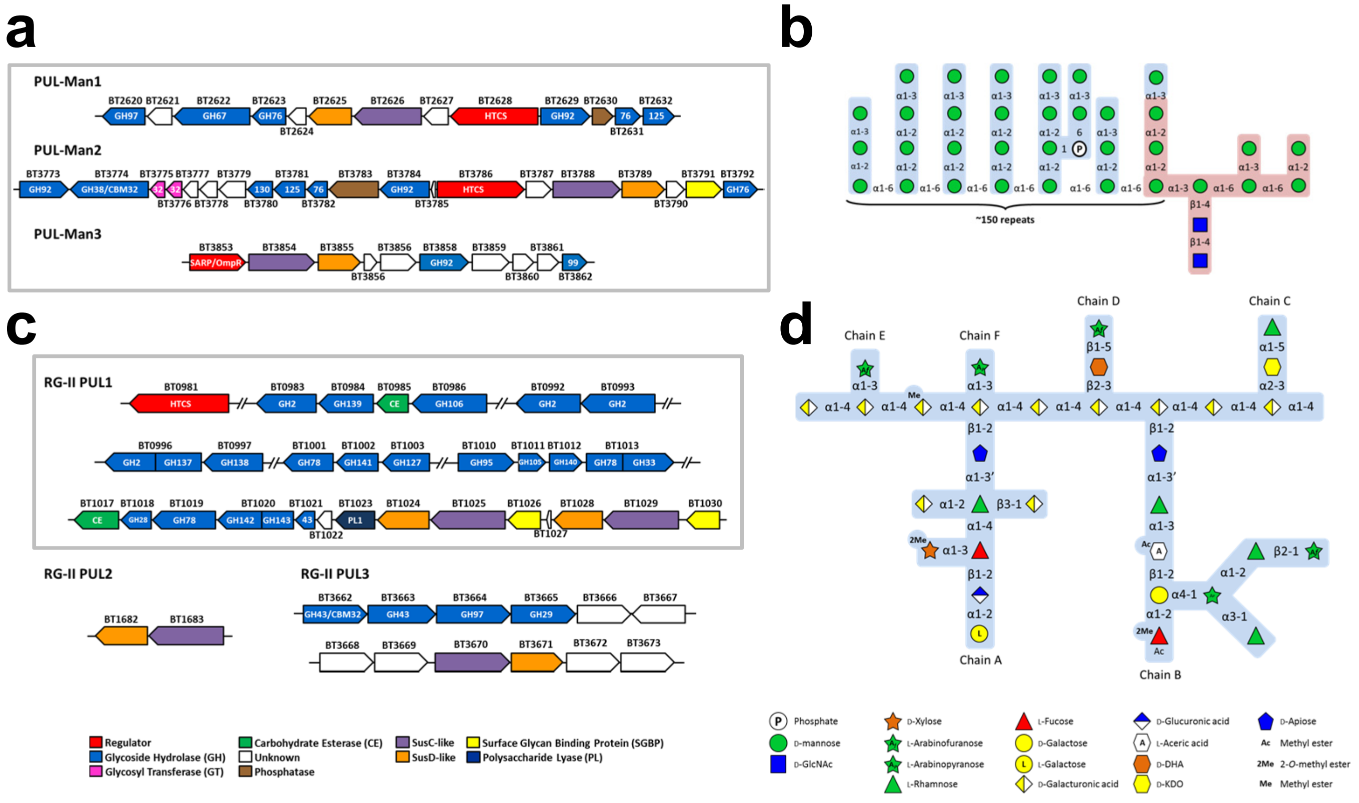

Supplementary Figure 1: (**a**) Structure of PUL-MAN1, 2 and 3, pathways responsible for the utilization of YM by *B. theta*. (**b**) Composition and linkage structure of YM. (**c**) Structure of PUL-RGII1, 2, and 3 responsible for the utilization of RGII by *B. theta*. Annotated genes and enzyme family are labeled. (**d**) Composition and linkage structure of RGII. Annotated genes and enzyme families are labeled. The gray boxes denote the genes that were removed by mutagenesis. Monosaccharides are depicted using the Consortium for Functional Glycomics nomenclature (18).
