# Supplementary material for: Single cell fluorescence imaging of glycan uptake by intestinal bacteria
Source: ISME J. 2019 Apr 1;13(7):1883–9. doi: 10.1038/s41396-019-0406-z (PMC6776043; doi:10.1038/s41396-019-0406-z)
Supplement: Supplementary file 4 — Sup2 [file 41396_2019_406_MOESM4_ESM.pdf]

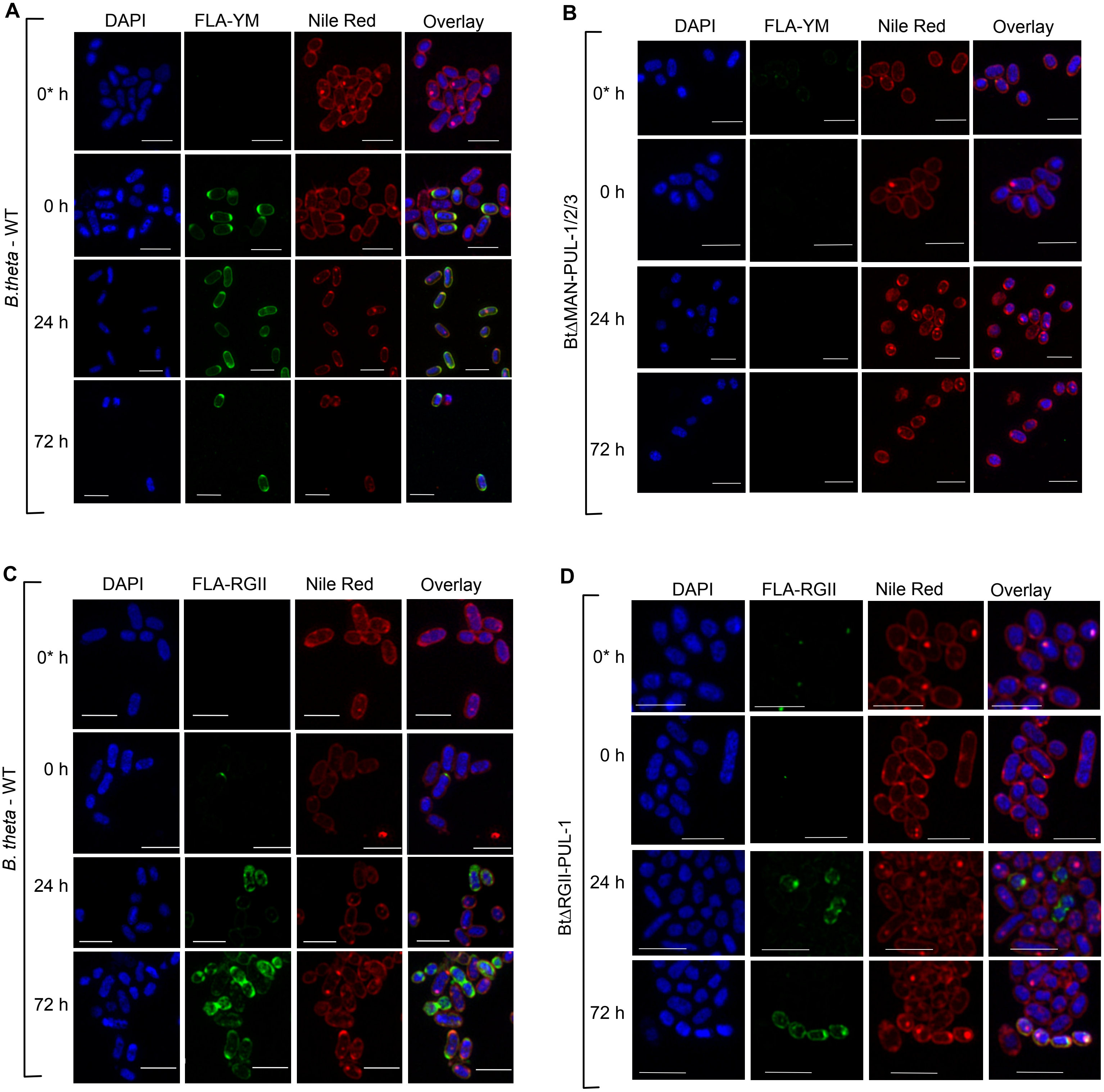

Supplementary Figure 2: Full panel display of fluorescently labeled *B. theta* mutant strains visualized by super-resolution structured illumination microscopy (SR-SIM). Cells are stained with DAPI (blue), FLA-YM or FLA- RGII (green), and Nile Red (red); and displayed at 0* (true zero), 0 (directly after glycan addition), 24 and 72 hours. (**A & B**) Wild-type *B. theta* and mutant BtΔMAN1/2/3 cells labeled with FLA-YM. (**C & D**) Wild-type *B. theta* and mutant BtΔRGII cells labeled with FLA-RGII. Size bars = 2 μM.
