# Supplementary material for: Single cell fluorescence imaging of glycan uptake by intestinal bacteria
Source: ISME J. 2019 Apr 1;13(7):1883–9. doi: 10.1038/s41396-019-0406-z (PMC6776043; doi:10.1038/s41396-019-0406-z)
Supplement: Supplementary file 5 — Sup3 [file 41396_2019_406_MOESM5_ESM.pdf]

A

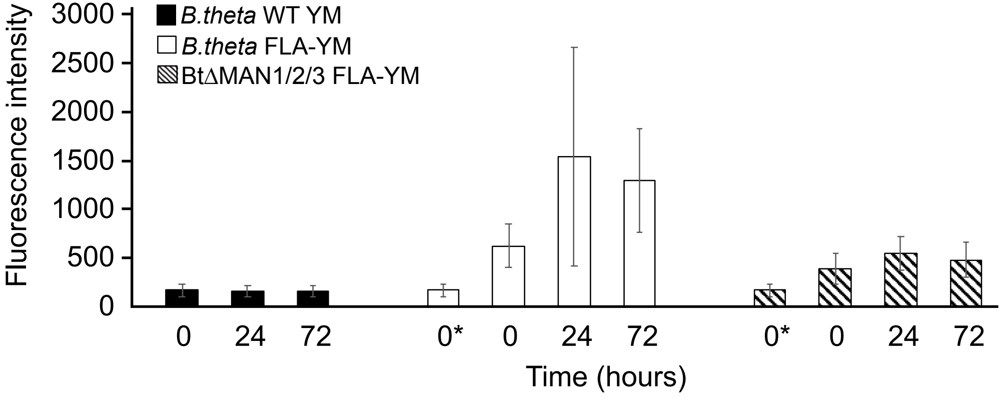

B

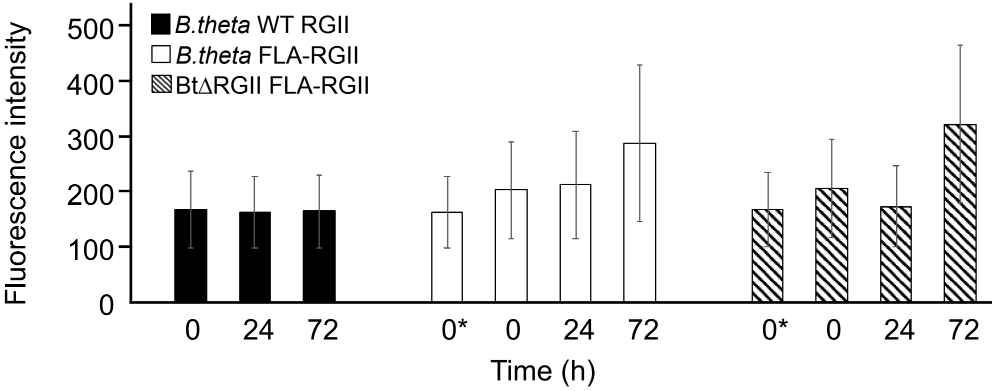

Supplementary Figure 3: Time-dependent uptake of fluorescent glycan conjugates by *B. theta*. **A**) Change in mean fluorescence intensity of *B. theta* (white) and BtΔMAN1/2/3 (dashed) incubated with FLA-YM and a control (*B. theta* incubated with unlabeled YM, black) over time (0* (true zero), 0 (directly after glycan addition), 24 and 72 hours). **B**) Change in mean fluorescence intensity of *B. theta* (white) and BtΔRGII (dashed) incubated with FLA-RGII and a control (*B. theta* incubated with unlabeled RGII, black) over time (0* (true zero), 0 (directly after glycan addition), 24 and 72 hours). N=8,500 and error bars = standard deviation.
